# Ambiguous Effects of Autophagy Activation Following Hypoperfusion/Ischemia

**DOI:** 10.3390/ijms19092756

**Published:** 2018-09-13

**Authors:** Michela Ferrucci, Francesca Biagioni, Larisa Ryskalin, Fiona Limanaqi, Stefano Gambardella, Alessandro Frati, Francesco Fornai

**Affiliations:** 1 Department of Translational Research and New Technologies in Medicine and Surgery, University of Pisa, Via Roma 55, 56126 Pisa, Italy; michela.ferrucci@med.unipi.it (M.F.); larisa.ryskalin@unipi.it (L.R.); f.limanaqi@studenti.unipi.it (F.L.); 2 IRCCS Neuromed, Via Atinense 18, 86077 Pozzilli (IS), Italy; francesca.biagioni@neuromed.it (F.B.); stefano.gambardella@neuromed.it (S.G.); alessandro.frati@uniroma1.it (A.F.)

**Keywords:** cerebral blood flow, hypoxia, starvation, brain ischemia, autophagy, mitophagy, neurodegeneration

## Abstract

Autophagy primarily works to counteract nutrient deprivation that is strongly engaged during starvation and hypoxia, which happens in hypoperfusion. Nonetheless, autophagy is slightly active even in baseline conditions, when it is useful to remove aged proteins and organelles. This is critical when the mitochondria and/or proteins are damaged by toxic stimuli. In the present review, we discuss to that extent the recruitment of autophagy is beneficial in counteracting brain hypoperfusion or, vice-versa, its overactivity may per se be detrimental for cell survival. While analyzing these opposite effects, it turns out that the autophagy activity is likely not to be simply good or bad for cell survival, but its role varies depending on the timing and amount of autophagy activation. This calls for the need for an appropriate autophagy tuning to guarantee a beneficial effect on cell survival. Therefore, the present article draws a theoretical pattern of autophagy activation, which is hypothesized to define the appropriate timing and intensity, which should mirrors the duration and severity of brain hypoperfusion. The need for a fine tuning of the autophagy activation may explain why confounding outcomes occur when autophagy is studied using a rather simplistic approach.

## 1. Introduction

Autophagy (ATG) and ubiquitin proteasome system (UPS) are recruited for protein and organelle degradation within eukaryotic cells. It is commonly believed that ATG and UPS possess distinct substrates and act independently from each other within segregated cell compartments. While UPS degrades short-lived proteins, ATG clears long-lived proteins and cell organelles, such as mitochondria, through their delivery to late endosomes and/or lysosomes [1]. A number of conditions may be counteracted by the ATG activation, such as age-related telomere and genomic alterations, loss of proteostasis, dysregulation of nutrient-sensing pathways, mitochondrial dysfunctions, limited regenerative ability, and altered cell to cell communication [2,3,4,5].

**Figure 1:**
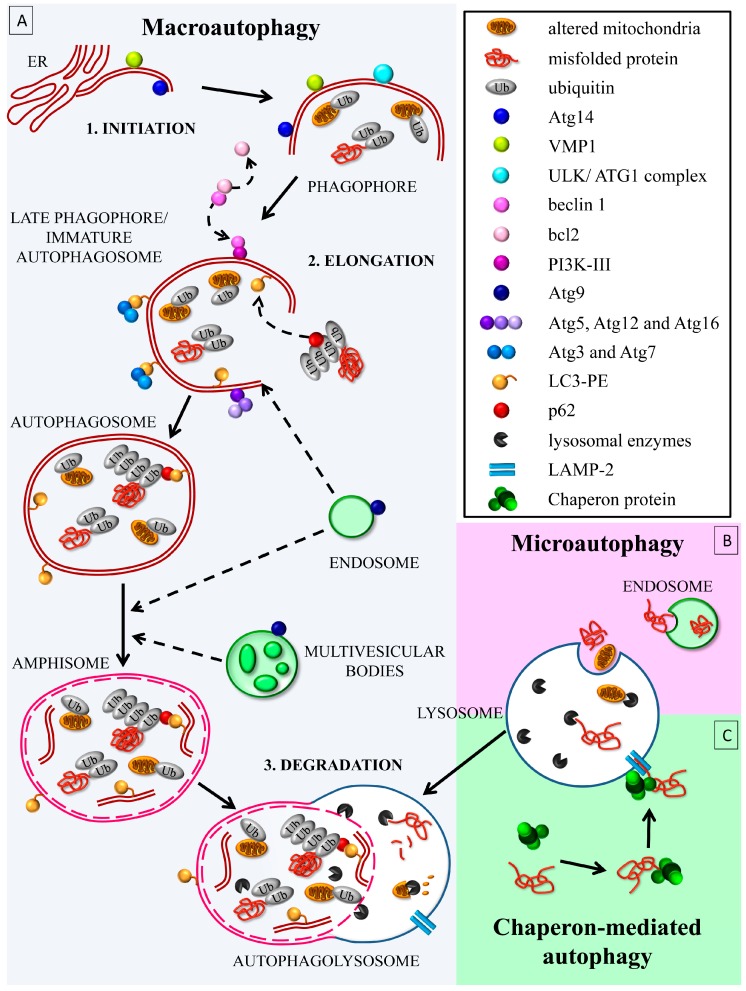
The autophagy (ATG) clearing pathway. (**A**) Macro-ATG follows the activation of the autophagy initiation complex, named the ATG1 or Unc-51 like autophagy activating kinase (ULK) (Unc-51 like autophagy activating kinase) complex. This leads to the phagophore, an incomplete double-membrane structure, which gems from the mitochondrial or endoplasmic reticulum carrying Atg14 and vacuolar membrane protein 1 (VMP1). The ULK/ATG1 complex phosphorylates Atg14, allowing the recruitment of beclin 1 from bcl2 on the phagophore membrane. Beclin 1 binds to phosphatidylinositol-3-kinase class III (PI3K-III)/VPS34, forming the active complex PI3K-III, which produces a focal increase of phosphatidylinositol-3-phosphate (PI3P). This leads to the followng: (i) the Atg9-mediated recruitment of endosomes and multivesicular bodies, and the (ii) induction of ubiquitin-like reactions. These consist of the formation of the Atg5/Atg12/Atg16 complex; or the assembly of the E1-like Atg7-mediated and the E2-like Atg3-mediated conjugation of LC3 to the phosphatidyletanolamine (PE), thus forming LC3-II (also called LC3 lipidation). Meanwhile, the late phagophore folds in order to enwrap the aged or damaged cell components, such as the ubiquitinated mitochondria and p62-bound poly-ubiquitinated proteins, which are sequestered within a mature autophagosome. The fusion between the autophagosome with the lysosome involves proteins belonging to the soluble *N*-ethylmaleimide sensitive factor attachment protein receptors (SNARE) complex. (**B**) Micro-ATG is traditionally described as a direct transport of several substrates into lysosomes for degradation; recently, it has been associated with the delivery of cargoes to late endosomes/multivesicular bodies. This latter process is dependent from ESCTR and Hsc70 proteins. (**C**) Chaperone-mediated ATG is a chaperone-mediated binding of specific substrates, which are delivered to the lysosomes through a lysosomal receptor, LAMP-2. Solid arrow: main step, Dotted arrow: molecule/vesicle trafficking.

ATG degrades the cell components to produce energy as a natural response to cell starvation [6]. However, apart from extreme conditions, ATG is slightly active even in baseline cell physiology, in order to guarantee the turnover of various cell components, thus avoiding the accumulation of aged proteins or altered organelles. ATG machinery consists of a variety of membrane structures, which organize to form vesicles, where abnormal material is segregated and delivered to lysosomal degradation. Three distinct ATG mechanisms are described, (i) macro-ATG, (ii) micro-ATG, and (iii) chaperon-mediated ATG (CMA) (Figure 1).

Macro-ATG consists of autophagosomes. In the initiation phase, a double membrane-bound phagophore gems from the endoplasmic reticulum (ER) and/or mitochondria; during the elongation phase an immature autophagosome appears as an open membrane structure, named late phagophore, where the ubiquitin-bound complex starts to be hosted. Furthermore, the ceiling of the vesicle generates the mature autophagosome, a vesicle compartment, which includes a volume of normally electron-dense cytosol. A variety of ubiquitin-dependent mechanisms shuttle altered substrates within the autophagosome, thus increasing its electron-density.

During its progression, the autophagosome is driven along microtubules to merge with lysosomes, forming the autophagolysosome, which contains a variety of enzymes [7,8,9,10]. Each ATG phase is characterized by specific ATG markers, which bind to the membrane structures allowing for deciphering the specific step within the ATG flux [11] (Figure 1A).

Micro-ATG differs from macro-ATG as small volumes of cytosol are directly enwrapped into lysosomes [12]. This may occur through the enwrapping of cytosolic proteins in the lysosome (membrane invagination or membrane protrusion); alternatively, cytosolic proteins are delivered through the endosomal sorting complexes required for transport (ESCRT) once bound to chaperonine Hsc70 [13,14] (Figure 1B). In contrast, CMA works through the molecular interaction of chaperones with a lysosome receptor called lysosome-associated membrane protein-2 (LAMP-2), which allows a receptor-mediated translocation into lysosomes of specifically tagged proteins [15] (Figure 1C).

Only macro-autophagy requires a previous ubiquitination of the substrates. When ATG removes the mitochondria, the process is named mitophagy. Mitophagy is driven by the expression of mitochondrial PINK1 (PTEN induced putative kinase 1), which recruits parkin within the altered mitochondria [16]. Here, parkin promotes the binding of mitochondrial proteins to ubiquitin [17,18] in order to be shuttled to the autophagosome or a nascent phagophore. At least three mitochondrial proteins (mitofusin 1, mitofusin 2, and voltage-dependent anion-selective channel protein 1) undergo parkin-dependent ubiquitination [19]. The mitochondrial proteins BNIP3 (Bcl2/adenovirus E1B 19 kDa interacting protein 3) and BNIP3L/NIX (BNIP3 like), as well as the ATG receptor optineurin, are also key for mitophagy. These proteins drive the mitochondria towards the autophagosome through the binding of LC3 (microtubule-associated protein 1 light chain 3, MAP LC3-I) [20,21].

The alteration of ATG occurs during aging and in most age-related neurodegenerative disorders, including Parkinson’s (PD) and Alzheimer’s disease (AD), leads to the accumulation of damaged organelles and unfolded/misfolded proteins [2,3,6,22,23,24,25].

## 2. The Autophagy Pathway as a Hot Topic in Brain Hypoperfusion/Ischemic Research

Research on brain hypoperfusion has recently been increasing exponentially [26,27,28]. In fact, differing from stroke, which causes sudden brain damage, chronic brain hypoperfusion (CBH) is a slight and smoldering insult, produced by a moderate though persistent reduction in blood supply. This allows neural cells to organize adaptive responses, which might compensate cell functions for prolonged time intervals. This phase may last a variable time interval, depending on the severity of the hypoperfusion. In detail, brain hypoperfusion indicates a condition of reduced cerebral blood flow (CBF), which can even be long-lasting, and for this reason we usually refer to the term of CBH. In the early stage, CBH may be asymptomatic, although cognitive/executive impairment may occur, depending on the duration and severity of hypoperfusion, and the age of the subjects [28,29]. For instance, a mild-to-moderate reduction of CBF is common during aging and primarily affects the cerebral cortex and basal forebrain [30,31,32,33,34,35]. When it surpasses a critical threshold, hypoperfusion is critical for cell homeostasis [36,37], and it is associated with a decline in the cerebral metabolic rate for O_2_ (CMRO_2_) and glucose (CMRGl) [38,39,40,41]. If protracted over time, brain hypoperfusion causes a loss of energy and may produce neurological symptoms, often featuring cognitive impairment [27,42]. In line with this, CBH contributes to the key mechanisms generating vascular dementia [27], and it is a major risk factor for cognitive impairment [27,43,44,45,46].

It is generally assumed that in CBH, the reduction of CBF is moderate and may last for several years, and is asymptomatic at first, but progressively damages brain tissue. In contrast, brain ischemia is a sudden symptomatic acute event. When the term cerebral ischemia is used, it implies a severe occlusion of blood vessels, which develops suddenly and may recover clinically, such as in a transient ischemic attack or leading to cerebral infarction or sudden death, as it occurs in ischemic stroke [47]. Cerebral ischemia can be classified into three types, thrombotic, embolic, and hemodynamic, according to its etiology [48], and into two types, focal and global ischemia, based on the extent of the ischemic area [49]. During the maturation of irreversible ischemic brain damage, there is a necrotic area surrounded by ‘area penumbra’, where the surviving cells develop a number of alterations, which may be considered reminiscent of neurodegeneration though in a condensed time interval [50]. To remark such a condition, the term acute neurodegeneration can be used. On the other hand, CBH does not possess such a clear-cut pathological zoning, being a quite homogeneous region, which slowly develops cytopathological alterations in a longer time interval. Within this time frame, compensatory mechanisms concur with the deleterious deficit in the blood supply of O_2_ and nutrients to determine a pathological area, which markedly differs from that occurring following brain ischemia. Nonetheless, the reduction of cerebral oxygen supply is a common determinant of both brain ischemia and CBH. Hypoxia can also occur without a reduction of CBF, through other mechanisms such as reduced blood oxygenation, occurring in acute lung injury, or a decreased oxygen transport in anemia. Among the variety of molecular mechanisms, which may be involved in producing the tissue alterations following brain hypoperfusion/ischemia, it is worth mentioning a number of changes affecting the small cerebral vessels both in structure and density, with concomitant alterations in the blood brain barrier (BBB) integrity and impairment of the neurovascular unit [51,52,53,54,55]. When CBH occurs naturally or during experimental conditions, the BBB is altered [56,57,58] and neurovascular coupling is depressed. This is associated with cognitive impairment and neuropathology, mainly in the hippocampus [59,60,61]. This is concomitant with the accumulation of proteins such as β-amyloid and phospho-tau [62,63,64,65,66,67,68,69]. This confirms how CBH may trigger a cascade, which leads to neuronal degeneration [26]. The accumulation of pathological proteins involved in Alzheimer’s disease results, even following brain ischemia. In this respect, Pluta’s lab found that the amyloid protein precursor increases after the transient ischemia in both experimental animals and humans [70,71]. The defective proteolytic clearance of β-amyloid peptide due to the impaired ATG is found to be involved in extracellular amyloid deposition, thus contributing to Alzheimer’s disease pathogenesis [51]. Remarkably, the dysregulation of ATG genes following brain ischemia was found concomitantly with significant alterations in the expression of the amyloid precursor protein, secretases, as well as the presenilin 1 and 2 genes [72,73,74,75]. Moreover, the accumulation of the amyloid protein precursor correlates with decreased expression of caspase 3 [70,71], and the β-amyloid peptide has been found to potentiate the tau protein phosphorylation [76]. Overall, these findings suggest that the dysregulation of ATG- and apoptosis-related genes found in the post-ischemic brains of rats may enhance the ischemia-induced accumulation of Alzheimer-related proteins [74,75,77]. This strengthens the role of hypoperfusion/ischemia in the pathological protein deposition, leading to neurodegenerative dementia.

Being a molecular pathway, which is promptly activated upon starvation, oxidative stress, and hypoxia, ATG is now attracting much interest in ischemia/hypoperfusion research [74,75]. In this respect, it is well-known that ATG is markedly increased during the reperfusion phase, which follows an ischemic insult [78,79]. Furthermore, the authentic functional significance of this increased ATG activity is still under debate, and contradictory data are reported concerning its beneficial or detrimental effects [80,81,82,83]. ATG proteins and vacuoles increase concomitantly with the apoptotic markers within dying neurons. This supports the hypothesis for a detrimental role of ATG in post-ischemic cell death [80,84,85,86,87].

A genetic analysis of the specific ATG and apoptosis markers was carried out in rats by Pluta’s lab [74,75], following ischemia in medial temporal lobe and specifically within hippocampal CA1. These researchers found significant alterations of the expression of specific ATG-, mitophagy-, and apoptosis-related genes [74,75]. In particular, these findings suggest that the removal of damaged mitochondria by mitophagy and apoptosis are responsible for the neuronal death in the post-ischemic brain [75]. The involvement of ATG in endothelial damage leading to the impairment of BBB integrity during cerebral ischemia is still controversial and it is worth being further investigated [55]. On the other hand, during the post-ischemic reperfusion phase, ATG inhibition (i.e., by the constitutive activation of mammalian target of rapamycin (mTOR), administration of the ATG inhibitors, and Atg7 knockdown) increases the neuronal damage and cognitive impairment. This suggests a protective role of ATG during ischemia-induced brain damage [78,88,89,90]. In fact, the enhancement of ATG during post-ischemic reperfusion or before inducing a ischemic insult (the so-called ‘ATG pre-conditioning’), decreases the neuronal damage and reduces the neurological deficits [78,81,91,92,93,94,95,96,97,98,99].

CBH is much less investigated than ischemia. This is likely to depend on the smoldering clinical impact offered suddenly by CBH compared with stroke. Only a few manuscripts are available dealing with ATG and CBH, which does not allow for one to get a solid opinion whether it is protective or deleterious [67,86,100,101,102,103]. Therefore, the present review is thought to clarify the state of the art in such a quickly growing topic. In keeping with this, a big discrepancy exists between the amount of investigations about ATG in acute cerebral ischemia [104,105] and CBH [26,67,86,100,101,102,103]. These latter studies involve human patients and experimental models, which are reported in Table 1 [29,106,107,108,109]. It is important to emphasize that the choice of the appropriate experimental model and the quality of the experimental design are key in order to reproduce a long-lasting reduction of CBF, such as in CBH, or the more severe and sudden arrest of blood flow, such as in cerebral ischemia [110]. The experimental models of brain hypoperfusion/ischemia consist of a reduction of CBF, achieved by the mono- or bi-lateral occlusion of large cerebral arteries, thus resulting in a reduction of the blood flow mainly affecting the brain cortex and hippocampus [110]. The type, number, and duration of the cerebral vessel occlusion may determine the severity of the hypoperfusion. For instance, the model of two vessel occlusion, consisting in the bilateral common carotid artery occlusion, is performed so as to produce a mild reduction of CBF, according with CBH [29,106,111,112,113,114,115]. A more severe reduction of CBF, such as that occurring in cerebral ischemia, may be produced by increasing the number of vessels occlusion. For instance, the models of three or four vessel occlusions, such as the permanent bilateral occlusion of the vertebral arteries, followed by the transient bilateral occlusion of the carotid arteries [116], or the permanent occlusion of the middle cerebral artery, allows one to study the ischemic injury produced by a sudden and consistent reduction of CBF [117,118,119]. On the other hand, the vascular anatomy among mammals shows variations between species and even strains, with significant species-specific differences in the number of arterial collaterals, upon which the capacity to restore the blood flow after vessel occlusion depends [120]. For instance, to produce global brain ischemia in rats or mice, it is necessary to limit the blood flow in almost three or four arteries that supply the brain (namely, carotid and vertebral arteries), or to reduce the systemic arterial pressure concomitantly with the bilateral occlusion of the common carotid arteries [121,122]. In contrast, the bilateral occlusion of the common carotid arteries (two vessel occlusion model) is able to produce global cerebral ischemia in gerbils [123,124,125]. On the other hand, oxygen–glucose deprivation (OGD) is widely in vitro as a model for cerebral ischemia to elucidate cellular and molecular mechanisms [109,126]. By analyzing these models, a strong increase of ATG is consistently evident in both in vivo and in vitro. Interestingly, considering the in vivo models of CBH, several studies show the occurrence of a wide temporal gap between the ATG activation and the occurrence of cell death [103,127]. In fact, while ATG increases immediately after the induction of brain hypoperfusion, cell death appears several weeks after CBH induction [106]. During this phase, the cell metabolism is expected to be modified in order to cope with the mutated nutrient supplies. A description of the main signaling pathways involved in these CBH-induced metabolic changes may help to comprehend the scenario where ATG is activated in the course of CBH.

### 2.1. Effects of Chronic Brain Hypoperfusion on ATG Activity

**Figure 2:**
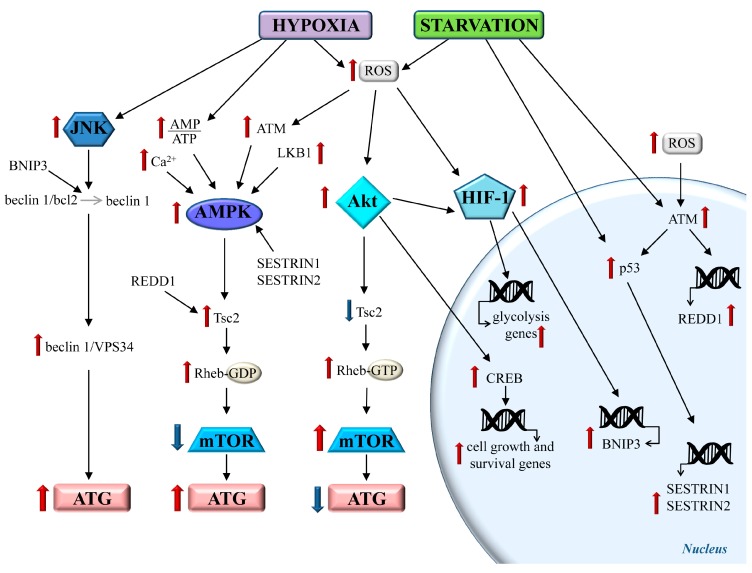
The main metabolic pathways triggered by chronic brain hypoperfusion (CBH). Hypoxia and starvation occurring during CBH activate several cell pathways. The AMP activated protein kinase (AMPK) pathway is strongly activated during CBH. Increased AMP binds to AMPK, which is phosphorylated by several kinases, such as LKB1. Glucose deprivation increases p53, which stimulates the transcription of sestrin 1/sestrin 2 proteins, which, in turn, enhance the AMPK activation. Reactive oxygen species (ROS)-induced DNA damage increases the ataxia-telengiectasia-mutated (ATM) activity, thus leading to increased p53 and REDD1 transcription. Upon oxidation by ROS, cytosolic ATM forms homodimers and activates AMPK by phosphorylation. CBH-induced calcium overload activate AMPK through calcium calmodulin-dependent kinases. AMPK inhibits mammalian target of rapamycin (mTOR) by acting on the Tsc2 protein. Tsc2 is part of the Tsc1/Tsc2 complex, which converts the guanosine triphosphate (GTP) to guanosine diphosphate (GDP)-bound Rheb, which causes the inhibition of mTOR. In particular, AMPK phosphorylates Tsc2 at two sites, thus enhancing the ability of the Tsc1/Tsc2 complex to block Rheb-dependent mTOR activation. Finally, mTOR inhibition leads to ATG activation. The hypoxia-induced Akt pathway stimulates mTOR by inhibiting Tsc2. This occurs through Akt-dependent phosphorylation of Tsc2, thus inducing its dissociation from the complex Tsc1/Tsc2. This stabilizes Rheb to activate mTOR, thereby inhibiting ATG. The activation of Akt also triggers a phosphorylation cascade, producing various effects. A key protein targeted by Akt is the transcription factor cyclic AMP-responsive element binding protein (CREB), which induces the expression of genes related to cell growth and survival, such as brain derived neurotrophic factor (BDNF) and the anti-apoptotic protein bcl2. Moreover, Akt increases the expression of the alpha subunit of the transcription factor HIF-1, which is essential for hypoxia-inducible factor-1 (HIF-1) transcriptional activity. In turn, HIF-1 regulates the transcription of the genes related to anaerobic metabolism, thus promoting the shift towards the glycolytic metabolism. CBH-induced hypoxia and starvation also activate ATG throughout mechanisms that do not involve mTOR. The hypoxia-induced HIF-1 increases the expression BNIP3, which, via beclin 1, promotes ATG. An increased amount of beclin1 occurs through the c-Jun N-terminal kinase (JNK) pathway under starvation and oxidative stress. Red arrow: increased activity/levels, Blue arrow: decreased activity/levels, Grey arrow: transition.

In the experimental model of CBH, the reduction of CBF is variable, depending on the specific model and time interval at which it is measured [29,106]. For example, in a two vessel occlusion (2VO) rat model, one week after the induction of CBH, the blood flow is reduced to roughly 30% within the isocortex and 20% within the hippocampus, compared with intact rats [106]. The reduction of O_2_ and nutrient supply causes a drop in the ATP levels and an increase of the reactive oxygen species (ROS). The main cell pathways, which are triggered under CBH, are schematized in Figure 2.

Hypoxia and starvation accompany CBH and induce substantial changes in cell metabolism. Decreased ATP levels increase the AMP/ATP ratio, which immediately activates the AMP activated protein kinase (AMPK), a sensor of energy status of the cell [128,129]. The AMPK activity is also increased by glucose starvation, with a mechanism, which involves the activation of the stress-induced p53, sestrin 1, and sestrin 2 proteins [130]. Following the ROS-induced DNA double-strand breaks, the nuclear ataxia-telengiectasia-mutated (ATM) kinase triggers a phosphorylation cascade, which leads to an increase in the expression and cytosolic translocation of p53 [131], which in turn activates AMPK by promoting its phosphorylation via the liver B1 kinase (LKB1) [129,132,133]. Moreover, the ROS-dependent oxidation of cytosolic ATM causes its dimerization and the activation of AMPK [131]. The activation of AMPK stimulates ATG through inhibiting the mammalian target of rapamycin (mTOR) (Figure 2). In this way, as mTOR inhibits ATG, CBH is expected to increase the ATG activity.

mTOR is a conserved serine/threonine kinase that phosphorylates several substrates involved in protein translation [134]. The activity of mTOR is regulated by a variety of signals, in particular, mTOR-dependent protein synthesis depends on the energy and nutrients’ availability, and for hypoxia, in baseline conditions, the mTOR activity tunes essential pathways for the cell growth and proliferation (protein and nucleotide synthesis, lipogenesis) [135], whereas in the conditions of hypoxia, the glucose and amino acid deprivation mTOR activity is inhibited [135,136,137].

AMPK inhibits mTOR by phosphorylating Tsc2 (tuberose sclerosis complex 2) [138,139,140] (Figure 2). The inhibition of mTOR through Tsc2 is also produced by the regulation of development and DNA damage response 1 (REDD1) protein, which is induced by hypoxia, via a mechanism involving increased ATM [136,141] (Figure 2). The hypoxia and increased ROS levels induce the activation of the Akt pathway, which phosphorylates the substrates involved in cell growth and survival [142]. Among these, the cyclic AMP-responsive element binding protein (CREB) is involved in this hypoxic-induced increased expression of protective molecules, such as brain derived neurotrophic factor (BDNF) and bcl2 [91,143,144,145,146,147]. Moreover, Akt inhibits Tsc2, leading to mTOR activation and ATG inhibition [83,139] (Figure 2). Therefore, in hypoxic/ischemic conditions, ATG activity is oppositely modulated by the AMPK and Akt pathways through mTOR [148], which in turn, may determine per se the inhibition of ATG via different mechanisms [83,149]. Moreover, CBH induces ATG through mTOR-independent mechanisms. For instance, hypoxia and ROS, either directly or through Akt activation, increase the levels of hypoxia-inducible factor-1 (HIF-1) [83,150,151,152], which stimulates gene transcription in order to promote anaerobic metabolism [153]. In fact, the shift from an oxidative to glycolytic metabolism guarantees at least a few ATP production during CBH [154] (Figure 2). Moreover, HIF-1 induces ATG by increasing the BNIP3 protein, which increases the levels of the beclin 1/VPS34 complex to initiate the ATG process [155] (Figure 2). A similar mechanism is shared by the c-Jun N-terminal kinase (JNK) signaling [156,157,158] (Figure 2).

**Figure 3:**
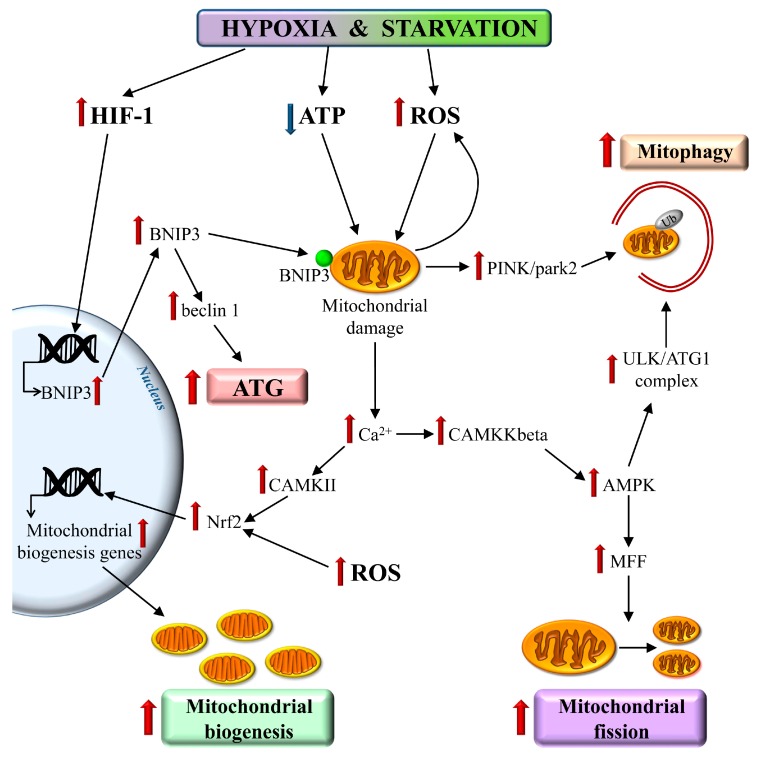
CBH induces mitophagy. Hypoxia heavily affects the mitochondria by interfering with the mitochondrial respiratory chain, and by dramatically decreasing the mitochondrial ATP production. In these conditions, dysfunctional mitochondria massively produce ROS to be released into the cytosol. Moreover, a loss of the mitochondrial membrane potential occurs. Thus, a mitochondrial depolarized membrane causes an inversion of calcium pumps and cytosolic calcium overload. The intracellular calcium activates the calcium-sensitive kinases, which activate AMPK. These calcium dependent kinases contribute to the activation of the Nrf2/SKN1 protein, which promotes the genes involved in mitochondrial biogenesis. The depolarized mitochondria are rapidly removed by ATG through the PINK1/parkin-dependent mitophagy. This is also induced by hypoxia-induced HIF-1. In fact, HIF-1 increases the BNIP3 protein, which acts on the mitochondrial membrane as a mitophagy receptor through its binding to LC3-II. Finally, the removal of dysfunctional mitochondria by mitophagy is also induced by AMPK-dependent ULK activation. Interestingly, the AMPK activation also promotes mitochondrial fission by enhancing the mitochondrial fission factor (MFF)activity. CAMKKbeta—calcium/calmodulin dependent protein kinase kinase beta; CAMKII—calcium/calmodulin dependent protein kinase type II. Red arrow: increased activity/levels, Blue arrow: decreased activity/levels.

Mitochondria are the organelles primarily affected during hypoxia. Several master regulators of the cell energy status, involving the HIF-alpha and AMPK, are found to determine the dramatic changes in the mitochondrial energy metabolism in order to promote glycolysis [159]. In this condition, the mitochondrial respiratory chain is markedly reduced, leading to metabolic uncoupling, reduced ATP, and a massive increase in ROS levels [160] (Figure 3). Dysfunctional mitochondria exhibit a depolarized membrane potential, which causes the inversion of calcium pumps and cytosolic calcium overload. The depolarized mitochondria are removed by ATG through the PINK1/parkin-dependent mitophagy [17,18]. In turn, the intracellular calcium concentration activates several calcium-sensitive kinases, which activate AMPK and then stimulates the AMPK-ULK-dependent mitophagy [129,161,162]. These conditions also promote the activation of Nrf2 (NF-E2-related factor 2)/SKN1 protein, which promotes the transcription of several genes involved in mitochondrial biogenesis [21]. Moreover, HIF-1 induces the mitochondrial removal by up-regulating the expression of BNIP3 [21,163] (Figure 3).

Interestingly, AMPK increases the mitochondrial number by stimulating the fission-dependent mitochondria duplication. In fact, AMPK phosphorylates the mitochondrial fission factor (MFF), which promotes the recruitment of dynamin related protein-1 (DRP-1). This localizes on the outer mitochondrial membrane, where it stimulates the mitochondrial fission [164,165] (Figure 3). Differently from mitochondria biogenesis, mitochondrial fission is a rapid, energy saving process, which allows damaged mitochondria to duplicate. The mitochondria generated by fission differ regarding the membrane potential. In fact, one of the two daughter’s mitochondria is depolarized. This allows damaged molecules to dilute differently in the two novel organelles, where the depolarized mitochondria are quickly removed by mitophagy. Recent studies report the occurrence of the cell-to-cell transmission of mitochondrial signaling (‘mitokines’), as well as the intercellular propagation of mitochondria through the tunneling nanotube, which may represent a way to widely diffuse the mitochondrial injury following hypoxia and oxidative stress [159]. Other compensatory responses are induced, including inflammation, unfolded protein response (UPR), and apoptosis. All of these increase under the hypoxic/ischemic insult [99,152,158,166,167,168,169]. In fact, hypoxia induces apoptosis and/or ATG. For instance, the JNK-dependent regulation of the bax-bcl2-beclin1 complex plays a dual role. In fact, JNK promotes th eATG activation through beclin 1, while it promotes apoptosis, inducing bax, and then caspase 9 and 3 activation [158,170,171]. This scenario is a paradigm for the need of a fine tuning, where slight dose differences may shift the balance between ATG and apoptosis during CBH in opposite directions [86,172]. Moreover, the induction of ATG needs to be finely registered in order to sort protective effects rather than producing ATG-induced neuronal damage.

### 2.2. Autophagy Modulation During Brain Hypoperfusion

Most of the pharmacological treatments known to be protective against neuronal damage induced by CBH are supposed to act as ATG inhibitors [86,103,128,173,174,175,176,177]. In particular, although characterized by different mechanisms of action, most reports emphasize the suppression of the ATG activation occurring in CBH [86,101,102,174,175,176,177]. This line of evidence suggests that the micro-balance working for ATG in CBH is shifted towards a deleterious effect of ATG on neuronal survival. This is the case of the L-calcium channel inhibitor nimodipine [103,128], the gamma-aminobutyric acid type B (GABAB) agonist baclofen [86], the anti-inflammatory and anti-oxidant agent lipoxin A4 methyl ester [173,174], the active alkaloid extract from *Leonurus cardiaca* leonurine [175], the endogenous cannabinoid system modulators WIN55,212-2 and URB597 [101,178], and the neurohypophyseal hormone arginine vasopressin [102]. In contrast, other studies provide evidence that in CBH ATG activation is protective for cell survival, whereas a detrimental effect is associated with the ATG reduction [67,100,102]. In fact, in experimental models of ischemia/hypoperfusion LC3-II levels are reduced, mTOR is activated and an increased expression and accumulation of the phosphorylated tau protein is documented [67,179,180,181]. In vitro experiments carried out in OGD conditions show that the classic ATG inducer rapamycin decreases cell death, while the ATG inhibitor 3-methyladenine (3-MA) and the lysosomal inhibitor MHY1485 increase cell death [182]. Thus, the dual (protective and detrimental) role of ATG remains unsolved so far.

In an effort to improve the analysis carried out on limited data regarding the effect of ATG in CBH, a validation of the methods used to monitor the ATG status is analyzed here. Most of the studies postulating the detrimental effects of ATG in CBH are grounded merely on the assay of ATG markers such as LC3 [86,101,103,127,174,183]. However, when the ATG flux is not progressing, LC3 is increased rather than suppressed, but ATG is not effective. In fact, when the ATG progression was specifically examined, a reduction rather than an increase of ATG was evident in CBH [100]. This is exemplified by two studies both reporting the neuroprotective effects of the L-type calcium channel antagonist nimodipine. This compound was reported to attenuate the excess of ATG in a rat model of CBH [103,127]. In fact, nimodipine rescued the spatial memory deficit and alleviated the neuronal damage in the cortex and hippocampal CA1 at two and four weeks after the induction of CBH [103]. An extended analysis up to eight weeks confirmed a long-lasting, the protective effect of nimodipine on cognitive functions and CA1 hippocampal neurons after induction of CBH [127]. Both of the studies correlated this neuroprotective effect with ATG inhibition, as nimodipine decreased the LC3-II levels [127] and the LC3-II/LC3-I ratio [103]. However, apart from measuring the ATG markers, these studies did not provide a direct assessment of the ATG flux, which is mandatory when inferring the number of markers as a measure of the ATG status.

Other studies show that the chronic treatment with URB597 (URB) carried out in a two vessel occlusion (2VO) rat model of CBH is protective against cognitive dysfunctions and hippocampal neuronal loss [101,178]. This was evaluated 12 weeks after the induction of hypoperfusion [101,178]. The CBH-induced neuronal damage was evaluated by the amount of the cell loss within the hippocampal CA1, which was significantly rescued by the URB post-treatment. Even this effect was associated with a reduction of the ATG markers, which were related to the mTOR activation. Unexpectedly, when the classic ATG inhibitor 3-MA was co-administered with URB, no further protection was observed [101]. In contrast, 3-MA worsened the cell damage [101]. Unfortunately, these authors failed to provide data measuring the effects produced by 3-MA on CBH [101]. In contrast with previous findings, a beneficial effect of a prolonged ATG activation in CBH was investigated in rats, where the molecular mechanisms underlying the neuroprotective effects of the arginine-vasopressin (AVP) neurohypophyseal hormone were documented [102]. This effect was produced through the stimulation of hippocampal vasopressin 1 (V1) receptors. In particular, the V1 activation enhances the CBH-induced ATG activation, as witnessed by the increased LC3-II/LC3-I and beclin 1 levels, as well as the LC3-II-positive puncta detected within the hippocampal neurons [102]. Moreover, in this study, an ultrastructural investigation showed that the autophagolysosomes were increased [102]. This latter finding, referred to four weeks after the induction of hypoperfusion, suggesting that the V1 stimulation was effective in promoting the ATG flux, which was instead impaired by CBH.

Evidence for the involvement of various ATG-related miRNAs in the pathophysiology of hypoxia-induced cell damage is increasing [184,185,186,187]. A careful analysis at the transmission electron microscopy allowed for correlating the specific intracellular ATG structures with the progression of the ATG flux during CBH [100]. This approach was integrated with immunocytochemical and immunoblotting investigations. This study demonstrates that during CBH, there is an increase of miRNA27a, which produces an inhibition of the ATG flux [100]. In fact, two weeks after CBH, when the increased expression of miRNA27a takes place, a fall in the ATG status occurred. This was evidenced by the profuse accumulation of the ATG vacuoles and increased p62 levels, along with high levels of LC3-II. Therefore, despite high levels of the classic ATG marker LC3-II, the ATG flux was depressed. In particular, miRNA27a reduces the level of the lysosomal receptor LAMP-2, thus suggesting a specific inhibition of the CMA-dependent lysosomal degradation [100].

The role of ATG in CBH remains very controversial [100,101,102,183]. This might depend on different experimental conditions in which the ATG pathway is evaluated in different studies, and the methods used to investigate ATG. In particular, in most studies, the ATG status was investigated only using the immunohistochemistry and/or immunoblotting assay for the ATG markers. No evidence was provided concerning the ultrastructural analysis of the ATG structures and the dynamics of ATG (for instance, through the measurement of the ATG substrates and/or the downstream molecules of the ATG inhibitor mTOR). In fact, the ATG markers do not necessarily relate to the ATG activity. As a paradigm is the case of the increased ATG markers occurring when the stagnant ATG vacuoles engulf the cells, as the ATG flux slows down [188]. This is not a pure theoretical detail, because a protective ATG should be an ongoing progressive flux [189]. In this respect, when the ATG flux was evaluated, a protective effect of ATG was found in CBH [100,101,102,182].

### 2.3. A Dynamic Hypothesis of the ATG Response in Chronic Brain Hypoperfusion

**Figure 4:**
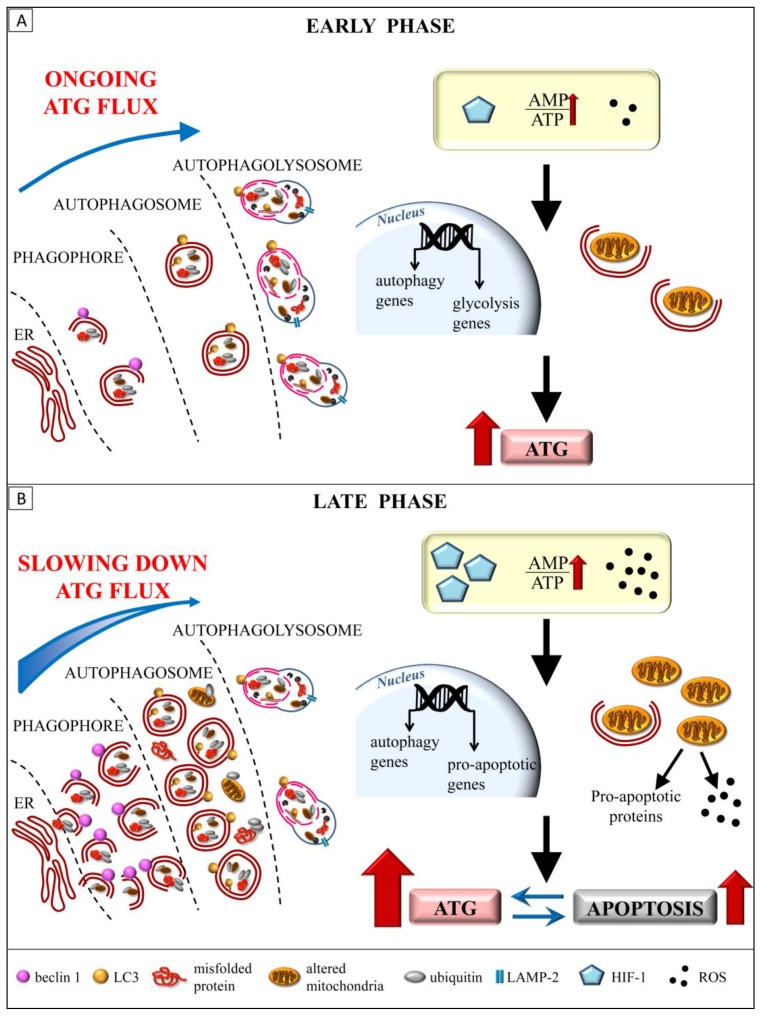
A dynamic model for the protective role of ATG in CBH. (**A**) The early phase of CBH is characterized by a sudden increase of the ATG activation. In this phase, the O_2_ and nutrients deprivation rapidly induce ATG, which is effective in the removal of altered cell components. Oxidative stress damages mitochondria, which are suddenly cleared by mitophagy. In this early phase of CBH, the ATG flux is normally ongoing and the ATG activation is key in delaying the cell death. (**B**) In the late phase of CBH, the protracted hypoxia, the severe energy deprivation, along with a further increase in the ROS levels, seriously worsen the cell functions. In these conditions, ATG further increased, but the broad cargoes overwhelm the ATG capacity, leading to a defective cell clearance. In particular, damaged mitochondria accumulate within the cytosol and become by themselves a source of additional ROS. Moreover, altered mitochondria release cytochrome c and other pro-apoptotic proteins. On the other hand, in this late phase of CBH, apoptosis is induced by a variety of stimuli. In particular, the apoptosis is triggered by the severe hypoxia, associated with mitochondrial damage and oxidative DNA injury. In this condition, a concomitant strong increase of both the ATG and apoptosis occurs. Moreover, the apoptosis and ATG are strictly interconnected and reciprocally regulated by several mechanisms. Therefore, the late CBH features as accumulation of ATG vacuoles, because of the relented ATG flux and increased apoptotic cell death. Red arrow: increased activity/levels, Blue straight arrow: modulatory effect, Blue curved arrow: intensity of ATG flux.

In the lack of a unitary view concerning which mechanisms are actually neuroprotective in CBH, here, we provide a hypothesis that tempts to reconcile the experimental data on the ATG flux during CBH with the CBH-induced metabolic changes, at different time intervals (Figure 4). Briefly, CBH represents a border-line condition, which put the viability of the neurons and glia at risk, without representing a frank toxic insult. In fact, a chronic reduction of CBF produces only mild-to-moderate hypoxia and starvation, compared with that occurring in stroke. However, in CBH, these conditions are long-lasting, and they may persist silent for years before producing brain damage. A mild but persistent hypoxic insult allows the cell to organize adaptive responses in order to compensate such critical conditions.

In our analysis, we keep the following two temporal phases during CBH distinct: (i) early phase, which starts soon after the onset of CBH, which lasts up to about one week (Figure 4A); (ii) late phase, when the cell metabolism is irreversibly impaired and the cell lost compensatory mechanisms lasting several weeks after the onset of CBH (Figure 4B).

In the early phase, the energy and hypoxia sensor systems are the main cellular pathways, which are promptly activated soon after the onset of brain hypoperfusion, leading to a switch from the oxidative-dependent aerobic metabolism to the glycolytic (anaerobic) metabolism. At the same time, in order to save energy and make cells less dependent on circulating substrates, the anabolic reactions, which for allow cell growth and proliferation, are markedly reduced, whereas the catabolism is enhanced. Within this context, early ATG activation induced by CBH should be interpreted as an immediate protective response (Figure 4A). Despite experimental models of CBH, the ATG activation is not investigated during the early hours of the induction of hypoperfusion, occurrence of increased ATG markers beclin 1, and LC3-II is widely documented within 24–48 h after a cerebral ischemic insult [74,81,190], thus suggesting a similar cellular response in CBH.

The exacerbation of cell damage obtained when ATG has been inhibited prior to, or immediately after the onset of ischemia/chronic hypoperfusion provides compelling evidence of ATG as being protective in delaying cell death in hypoperfusion [81,101,182]. On the other hand, long-lasting brain hypoperfusion leads to an unbalance cell homeostasis. In fact, when the reduction of blood flow is chronic, energy deficiency becomes progressively more severe, and so is oxidative stress, which damages the cell components. In this late phase, the ATG activity is further enhanced in an attempt to counteract the increasing levels of accumulating substrates. This corresponds to the so-called ‘excess’ of ATG, characterized by the highest levels of the ATG markers LC3-II and beclin 1, which are extensively documented in CBH [86,100,102,103,127]. In these conditions, apoptotic cell death is reported in CBH concomitantly with increased ATG markers [86], and treatments that reduce the ATG activation are found to be protective against a CBH-induced increase in the caspase level and/or neuronal death [86,103,127]. These findings suggest that ATG is responsible for the delayed cell injury observed in CBH.

However, an analysis of ATG flux at this late time interval (up to 12 weeks) reveals occurrence of defective ATG process. This may be explained by considering that, when hypoperfusion is long-lasting, the levels of the ATG substrates overwhelm the intrinsic capacity of the ATG machinery. As a matter of fact, a reduction of the autophagolysosomes and lysosomes compared with the autophagosomes witnesses for an engulfment of ATG, which occurs late during CBH [100]. This happens despite the high levels of LC3-II, which may disguise the investigator by suggesting an increased ATG activity, which does not take progress [86,100,103,127,183]. In fact, when the ATG substrates overwhelm the activity of the ATG pathway, then the elevated ATG markers co-exist along with a delayed progression of ATG; this appears morphologically as stagnant giant ATG vacuoles within the cytosol (Figure 4B). Such a chronic condition, which is likely to feature the late phase of CBH, is characterized by a complex cross-talk between ATG and apoptosis. In this respect, increasing evidence indicates that apoptosis and ATG are strictly interconnected, and multiple cellular pathways contribute to reciprocally regulate these events according to a fine balance [115,158,191,192,193]. For instance, it is documented that in hypoxic conditions, when mitochondrial damage and oxidative DNA injury become consistent, both ATG and apoptosis are triggered [115,191]. On the other hand, the apoptotic and ATG machineries share some regulatory signaling, which allows for switching from one to the other, depending on the metabolic state of the cells. In this respect, we reported the peculiar role of the beclin 1/bcl2 interplay in regulating ATG and apoptosis, thus allowing for turning ATG on or off, depending on the energy stores [158,171,194,195,196].

Independently from the pathway that is involved, in late CBH, a relented ATG, which causes an accumulation of damaged cell components, occurs together with an enhanced apoptosis, which depends on increasing pro-apoptotic stimuli. These mainly consist of damaged mitochondria, which are no longer removed by impaired ATG, and triggers the apoptotic cascade mainly through oxidative stress [115]. Therefore, even in the absence of a frank ATG suppression, later, in the CBH cell, death may derive from a loss of an effective ATG, which is bound to enhanced apoptosis.

In line with this, a recent review by Wolf et al. (2018) suggests that impaired ATG flux and lysosomal function, leading to increased autophagosome formation without progression towards autophagolysosomes, is critical in producing cell injury associated with ischemic insult [105]. Remarkably, these authors propose the term ‘autosis’, first coined by Liu et al. (2013) to indicate a form of ATG cell death with “unique morphological features, including increased autophagosomes/autolysosomes…, which occurs during treatment with autophagy-inducing peptides, starvation, and cerebral hypoxia–ischemia” [197]. This is supported by a number of studies dealing with ATG in acute brain injury, which consistently show that the treatments that enhance ATG by stimulating the ATG flux reduce the rate of autophagosome formation and are neuroprotective in experimental models of acute brain injury.

## 3. Conclusions

The present review represents an effort to disambiguate the role ATG in CBH compared with ischemia, as it appears from the recent literature. We show that ATG is markedly induced by CBH, which in fact produces an early and persistent increase in the ATG markers. However, the ATG flux, which reflects the actual ATG activity, exhibits a different time course, being increased at early time intervals after the onset of hypoperfusion, but being decreased when the hypoperfusion is long-lasting. In this respect, the increased amount of ATG vacuoles engulfing the cytosol of dying neurons might represent an epiphenomenon, which accompanies cell death in the late phase of CBH. A similar role of ATG, though in a condensed time interval, appears to occur in brain ischemia.

This latter point sheds a new light on the significance of the ATG activation during CBH, which dynamically develops through time, and might be susceptible to different modulations at different time intervals.

We propose that the correct tuning of ATG during CBH should be finely tuned in time and intensity, so as to produce beneficial effects in CBH.

## Figures and Tables

**Table 1:** Experimental models of brain hypoperfusion/ischemia.

**IN VIVO**			
**Model**	**Animal Species**	**Pathology**	**References**
2VO: BCCAo	Rat Mouse	Subcortical white matter lesions, and cortical and hippocampal damage	[106,107]
1VO: MCCAo	Mouse	No hippocampal damage at seven days	[108]
3VO: BCCAo + MVAo	Rat	Severe cortical and hippocampal lesions	[29]
**IN VITRO**			
**Model**	**Cell Culture**	**Culture Conditions**	**References**
OGD	Cell lines Primary neuronal cultures Brain organotypic cultures	Glucose-free medium under a deoxygenated atmosphere (hypoxic chamber)	[109]

BCCAo—bilateral common carotid artery occlusion; MCCAo—monolateral common carotid artery occlusion; MVAo—monolateral vertebral artery occlusion; OGD—oxygen–glucose deprivation VO—vessel occlusion.
